# Trends in Postneonatally Acquired Cerebral Palsy: Insights From a CP Surveillance Network

**DOI:** 10.1111/ppe.70004

**Published:** 2025-02-18

**Authors:** Hayley Smithers‐Sheedy, Sarah McIntyre

**Affiliations:** ^1^ Cerebral Palsy Alliance Research Institute, Specialty of Child & Adolescent Health The University of Sydney Sydney Australia

Cerebral palsy (CP) is a heterogeneous group of disorders caused by injury to the developing brain. The causal pathways to brain injury and the resulting CP motor type are varied and complex. Within the CP landscape, many small aetiological subgroups require investigation not only to identify potential opportunities for prevention but also to evaluate the impact of interventions targeting aetiologically relevant risk factors. In recent years, it has been encouraging to see increased attention on the classification, aetiology [1] and temporal trends [2] for children with CP in high‐income countries, who sustain a brain injury after the neonatal period and before 2 years of age, that is, those with postneonatally acquired CP (PNN‐CP).

In this issue of *Paediatric and Perinatal Epidemiology*, Delobel‐Ayoub and colleagues, using the Surveillance of Cerebral Palsy in Europe (SCPE) database [3], observed an average 2% annual decline in PNN‐CP between 1976 and 2012. As noted by the authors, the Australian Cerebral Palsy Register (ACPR) group is currently in the process of harmonising data fields for PNN‐CP to match the postneonatal classification system used by SCPE [1]. Even with current differences in postneonatal cause classification between networks, it is still possible to note the similarities in declining trends in PNN‐CP across Europe 1976–2012 and Australia 1973–2012 (7%–12% per five‐year epoch) [4, 5]. Across both networks, we have also observed declining trends of CP caused by postneonatal infections, likely reflecting the implementation of vaccines and improved management of children with common illnesses such as gastroenteritis [5].

Delobel‐Ayoub et al.'s findings also support observations made previously that children with PNN‐CP generally have more severe disabilities compared with children with pre/perinatally acquired CP [4, 6]. Further, the authors have identified that the most severe functional limitations in the PNN‐CP group were experienced by children with postneonatal aetiologies classified as hypoxic (e.g., near‐drowning, cardiac arrest), many of whom had grey matter injuries. Identifying public health and clinical strategies for primary and secondary prevention of these and other postneonatal aetiologies should be prioritised and paired with robust evaluation to guide public health and clinical interventions internationally [6]. Examples of this include targeted efforts to maximise infant vaccinations, and the introduction of pool fencing legislation, along with increased water safety campaigns which have led to documented declines in infectious disease and drownings [6].

The findings by Delobel‐Ayoub et al. and studies on CP prevalence and aetiology trends are only possible due to the support of families who allow their children's data to be included in CP registers and the dedicated efforts of clinicians and researchers maintaining these registers. Further, CP register networks bring together data from across geographical regions, for example, SCPE—Europe, ACPR—Australia, and the Global Low‐ and Middle‐Income Cerebral Palsy Register (GLMCPR) currently 15 countries and growing. These networks are valuable epidemiological resources, offering the power to study small subgroups, as demonstrated by Delobel‐Ayoub et al. for children with PNN‐CP. To further leverage the strength of these valuable resources, collaborations between registers/surveillance networks can provide additional power for small subgroups. Recent examples of powerful collaborations combining European and Australian data include research investigating CP and higher order multiples [7] and congenital anomalies [8]. Similarly, the GLMCPR has transformed our understanding of CP outside high‐income countries by comparing CP data from multiple low‐ and middle‐income countries [9], which can now be incorporated into global efforts to understand prevalence and trends [2].

Looking forward, individual CP registers and/or register networks, in collaboration with people with lived experience of CP, clinicians and researchers, can contribute to a global collaborative effort to map aetiological risk factors for CP using directed acyclic graphs (DAG) [10]. These hypothesis‐driven DAGs will provide a visual representation of causal relationships between variables and allow us to map the complex aetiological pathways to CP, revealing new opportunities for prevention. It is envisaged that causal DAGs for CP will be continually revised and updated to reflect contemporary research findings. A DAG, or potentially multiple DAGs, for the PNN‐CP group, would be particularly interesting, considering that many children in this group have pre/perinatal, sociodemographic and environmental factors contributing to their postneonatal brain injury and subsequent CP motor disorder. One example of this would be the case of children who have congenital anomalies due to maternal health conditions, genetics and/or environmental causes, who go on to experience a postneonatal brain injury resulting from surgery undertaken to repair the congenital anomalies. Understanding and mapping these causal pathways from preconceptional factors through the pre/perinatal period and into the postneonatal period in both high‐ and low‐ and middle‐income settings will be essential to identify risk reduction and prevention opportunities.

## Author Contributions

H.S.S. and S.M. contributed to the drafting of the work and approved the final version for publication. All authors agree to be accountable for all aspects of the work.

## Conflicts of Interest

The authors declare no conflicts of interest.

## Data Availability

Data sharing not applicable – no new data generated.
